# Druggable cavities and allosteric modulators of the cell division cycle 7 (CDC7) kinase

**DOI:** 10.1080/14756366.2024.2301767

**Published:** 2024-01-11

**Authors:** Elisa Rojas-Prats, Loreto Martinez-Gonzalez, Carmen Gil, David Ramírez, Ana Martinez

**Affiliations:** a Centro de Investigaciones Biológicas -Margarita Salas-CSIC, Madrid, Spain; b Centro de Investigación Biomédica en Red de Enfermedades Neurodegenerativas (CIBERNED), Instituto de 13 Salud Carlos III, Madrid, Spain; c Departamento de Farmacología, Facultad de Ciencias Biológicas, Universidad de Concepción, Concepción, Chile

**Keywords:** CDC7, DBF4, allosteric modulator, virtual screening, binding pockets

## Abstract

Cell division cycle 7 kinase (CDC7) has been found overexpressed in many cancer cell lines being also one of the kinases involved in the nuclear protein TDP-43 phosphorylation *in vivo*. Thus, inhibitors of CDC7 are emerging drug candidates for the treatment of oncological and neurodegenerative unmet diseases. All the known CDC7 inhibitors are ATP-competitives, lacking of selectivity enough for success in clinical trials. As allosteric sites are less conserved among kinase proteins, discovery of allosteric modulators of CDC7 is a great challenge and opportunity in this field.

Using different computational approaches, we have here identified new druggable cavities on the human CDC7 structure and subsequently selective CDC7 inhibitors with allosteric modulation mainly targeting the pockets where the interaction between this kinase and its activator DBF4 takes place.

## 1. Introduction

The cell division cycle 7 kinase (CDC7) is a serine/threonine kinase, firstly discovered in yeast^1^, that plays an important role in DNA replication and cell cycle progression in eukaryotic organisms^2^. Its kinase activity is regulated by its direct interaction with an activation subunit called DBF4, forming an activation complex known as DBF4 Dependent Kinase (DDK)^3^.

CDC7 has been found overexpressed in many cancer cell lines and certain primary tumours like ovarian, breast, liver, colon cancer, lung adenocarcinoma and melanoma^4–6^. For this reason, it has become of great interest as a new therapeutic target for cancer therapy, leading to the development of several inhibitors in pre-clinic and clinical trials^7^. Recently, CDC7 has also emerged as a valuable target to use in synergistic treatment with chemotherapy in the malignant small-cell lung cancer (SCLC)^8^.

Although its primary substrate is the minichromosome maintenance protein complex 2–7 (MCM2-7), a DNA helicase essential for DNA replication^9^. CDC7 has been also found to phosphorylate the transactive response DNA binding protein of 43 kDa (TDP-43), leading to its aggregation and accumulation in the motor neurons of some amyotrophic lateral sclerosis (ALS) and frontotemporal dementia (FTD) patients^10^. The relevant role of targeting CDC7 as a therapy for ALS and FTD has been recently reported with brain permeable inhibitors^11^^,^^12^, which points to the value of CDC7 inhibitors for the therapy of these devastating neurodegenerative diseases.

**Figure 1.**
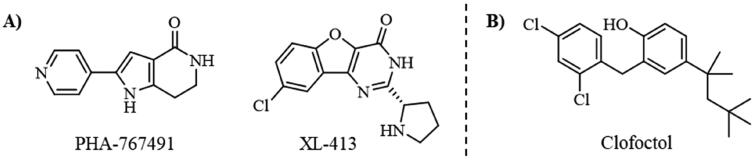
Some knowing CDC7 inhibitors A) ATP-competitives inhibitors and B) allosteric modulator.

To date, all the CDC7 inhibitors synthesised and characterised in the literature have been reported as ATP-competitive inhibitors: they interact with the catalytic site of the protein, which is mostly conserved in the different proteins from the human kinome. Two of the most studied inhibitors, known as PHA-767491 and XL-413, are depicted in Figure 1^13^^,^^14^. As result, most of these ATP-competitive inhibitors lack of selectivity against its specific target leading to non-desired secondary effects and unfortunately, without good effect on clinical trials. In this context, the discovery of allosteric modulators of CDC7 is a great challenge and opportunity in the field. In general, the allosteric sites are less conserved among kinase proteins, which allows activate or inhibit the kinase activity in a selective way without affecting other kinases^15^.

In the last decades, the development of allosteric modulators has considerably increased. To date, 6 allosteric inhibitors, included the recently discovered Asciminib^16^, have been approved by the Food and Drug Administration (FDA) for the treatment of different pathologies^17^ and more than a dozen are in clinical trials for the treatment of tumors^18^.

Recently, some molecules from the FDA chemical library, included the antimicrobial agent clofoctol (Figure 1), were identified to target allosterically CDC7 using a bioluminescent assay in cells^19^. These compounds were able to selectively inhibit CDC7 by interrupting the protein-protein interaction between this kinase and its activator (DBF4), showing to inhibit the phosphorylation of MCM2 on OEC-M1 oral cancer cell line, blocking the DNA replication and delaying cell cycle progression.

Apart from them, there is no other allosteric modulators of CDC7 reported in the literature. Thus, the search of new druggable cavities different from the catalytic site together with the discovery of new molecules able to bind this kinase with allosteric modulation will be an interesting approach for the development of more effective treatments not only for cancer, including SCLC, but also for ALS and other related TDP-43 proteinopathies.

Considering this background, the main objective of this work is to identify new allosteric binding sites on the surface of CDC7 and discover new allosteric modulators as a new and powerful strategy to regulate protein function for the treatment of cancer and neurological conditions. For this purpose, different computational approaches were used to identify new druggable cavities on the human CDC7 structure. Furthermore, virtual screening of our Medicinal and Biological Chemical (MBC) library^20^ was used to identify new selective CDC7 allosteric modulators.

## Materials and methods

### Pocket search

In order to identify new cavities on CDC7 surface, the fpocket software was used^21^. Fpocket is an open source protein pocket detection software that is freely available for download at http://www.sourceforge.net/projects/fpocket.

In first place, four CDC7 crystal structures (PDB codes: 4F99, 4F9A, 4F9B and 4F9C)^22^ were downloaded from the Protein Data Bank (www.pdb.org) and prepared using Maestro Protein Preparation Wizard tool^23^^,^^24^, removing all water molecules, ligands, and metal ions while adding missing hydrogens and ionising residues to pH 7.5. In the same way, the structure of the kinase activator (DBF4) was also removed. Then, an energy minimisation of the resulted structures was carried out using the OPLS-3 force field^25^. Once the different structures were prepared, they were subjected to pocket search using fpocket. Upon fpocket analysis, the four structures were analysed by visual inspection together with the pocket score values in order to identify conserved pockets among the different CDC7 structures as well as the residues involved.

### Ligand preparation

Prior to virtual screening, our Medicinal and Biological Chemistry (MBC) library^20^ was prepared using the LigPrep tool from the Schrödinger software package. LigPrep allows ligand preparation by adding hydrogen atoms, neutralising charged groups and generating the different ionisation states and possible tautomers followed by energy minimisation using the same force field previously used for protein preparation. All compounds were prepared at physiological pH conditions (pH 7.5).

### Virtual screening

Virtual screening of the MBC library was performed by multiple molecular docking campaigns using the Glide software through Maestro interface^26^^,^^27^. The binding site of the different ligands was defined by creating a grid box centred on pockets 1, 2 and 6 of the 4F9A structure which was previously prepared using Protein Preparation Wizard tool. The 4F9A crystal structure was selected to conduct the studies because the two cavities that comprise pocket 6 were completely defined in this structure after fpocket search. The docking studies of the different compounds were carried out with the Standard Precision (SP) Glide mode followed by a post-docking minimisation on all poses generated using the OPLS3 force field prior to the assignment of docking scores, leading/obtaining the top 10 poses per compound. Then, ligand poses with the highest ranked docking scores were selected (50%) and a new docking was performed this time with the Extra Precision (XP) scoring function, leading once again to 10 poses per compound state.

For pocket 6, the docking was performed ensuring the entire cavity was included in the gridbox. Furthermore, residues Thr109 y Cys123 were selected as key residues for the interaction filtering.

### Binding free energy calculations

The molecular docking results were refined by Molecular Mechanics-Generalised Born Surface Area (MM-GBSA) calculations of the different poses per ligand using Prime^24^. Residues within 5 Å distance of the ligands were treat flexible and the VSGB solvation model was used. This computational method combines molecular mechanics energy and implicit solvation models to rescore docking results and correlate the experimental activities (IC_50_) of the different compounds with their predicted binding energies (ΔG_bind_) against the protein^28^. The binding free energies between the ligands and the protein (CDC7) were calculated using the following equations:

(1) $$\Delta G_{\text{bind}}=\Delta \text{H }–\text{ T}\Delta \text{S }\approx \Delta E_{\text{MM}}+\Delta G_{\text{sol}}–\text{ T}\Delta S$$

(2) $$\Delta E_{\text{MM}}=\Delta E_{\text{internal}}+\Delta E_{\text{Coulomb}}+\Delta E_{\text{vdW}}$$

Where ΔE_MM_ includes internal (ΔE_internal_) (bond, angle and dihedral energies), electrostatic (ΔE_Coulomb_) and van der Waals (ΔE_vdW_) energies changes; ΔG_sol_ is the solvation free energy change expressed as the sum of the electrostatic solvation energy or polar contribution, and non-electrostatic solvation energy (ΔG_PB/GB_ + ΔG_SA_). Finally, TΔS is the change of the conformational entropy at a specific temperature; this contribution was not included as binding free energy calculations were used in this study to rescore docking results, and it has been suggested that the entropy term does not improve the results of MM-GBSA binding free energy calculation^29^^,^^30^.

Results were analysed by visual inspection considering docking score values and binding free energies (ΔG_bind_) against the different CDC7 pockets. Compounds with better ΔG_bind_ against pocket 1 (catalytic site) *versus* pockets 2 and 6 were discarded. This same protocol was applied to CDC7 known ATP-competitive inhibitors (PHA-767491 and XL413)^22^ and the allosteric inhibitor (clofoctol)^19^ described in the literature as reference.

### *In vitro* kinase experiment

Kinase-Glo® luminescent kinase assay was used to screen compounds for activity against CDC7. Human recombinant full-length CDC7/DBF4 kinase, substrate PDKtide (KTFCGTPEYLAPEVRREPRILSEEEQEMFRDFDYIADWC) and reaction buffer was purchase from Promega (CDC7/DBF4 Kinase Enzyme System, Cat#: V5088, Promega Biotech Ibérica, SL). The enzymatic reaction was performed in a total volume of 40 µL in white 96-well plates. In a typical assay, 10 µL of test compound at a final concentration of 10 µM, 10 µL of enzyme solution (25 ng/well), 10 µL of ATP solution (1 µM/well) and 10 µL of PDKtide substrate (0.2 µg/µL/well) were added to each well. The final DMSO concentration in the reaction mixture did not exceed 1%. After 60-min shake-incubation period, the enzymatic reaction was stopped with 40 µL of Kinase-Glo reagent (Kinase-Glo® Luminescent Kinase Assay kit, Cat#: V6711, Promega Biotech Ibérica, SL). Glow-type luminescence was recorded after 10 min using a GloMax® Discover Microplate Reader (Promega Biotech Ibérica, SL). The activity is proportional to the difference of the total and consumed ATP. The inhibitory activities were calculated based on maximal activities measured in the absence of inhibitor.

### Cell culture

The human neuroblastoma SH-SY5Y cell line was grown in DMEM media (Cat#41965039 Gibco/Thermo Fisher, Waltham, MA, USA) containing 10% (v/v) Foetal Bovine Serum (Cat#: F7524, Merck, Madrid, Spain) and 1% penicillin/streptomycin (Cat#15140–122, Gibco/Thermo Fisher Waltham, MA, USA). Cells were culture in a humidified 5% CO_2_ atmosphere at 37 °C.

### Evaluation of cell cycle divisionphases

Cells were seeded at initial concentration of 500.000 cells/well in a 6-well plate. After 24 h cells were treated with Clofoctol and potential CDC7 allosteric modulators at a concentration of 10 µM. The treated cells were pulsed with 20 μM BrdU in culture medium 4 h after compounds treatment. 24 h later cells were fixed and dual-stained BrdU (Y-axis) and 7-AAD (X-axis) prior to flow cytometry analysis was performed following the manufacturer’s instructions (BD Pharmigen^TM^ BrdU Flow Kit, Cat#: 559619). Flow cytometry analysis was done using CytoFLEX cytofluorimeter and CytExpert software (Beckman Coulter).

### Statistics

Statistical analyses were performed using Graph Pad Prism software version 6 (La Jolla, CA, USA). All the data are presented as mean ± standard error of the mean (SEM). Statistical significance was estimated using two-tailed Student’s t-test for comparisons between groups, or one-way ANOVA followed by the Bonferroni test for multiple comparations. A ‘*p* values < 0.05’ was considered statistically significant (*p < 0.05, **p < 0.01, ***p < 0.001, ****p < 0.0001).

## Results and discussion

### Pocket finding

In the last decades, different computational programs have been developed to determine druggable cavities on proteins surface based on different algorithms^31^. In this work, fpocket was selected for the identification of new cavities on the surface of CDC7. Fpocket is an open-source program for cavity detection based on α-spheres^21^. A α-sphere is a four atoms sphere at the surface of the protein, with no atom inside. In this sense, sites with a great number of closed spheres are identified as pockets, discarding those ones made of too small or large spheres. Furthermore, fpocket shows some information about residues involved and score the pockets based on different properties as hydrophobicity or size. Thus, pockets are numbering from 1 to *n*, being pocket 1 the catalytic site which use to be the most druggable pocket.

To perform fpocket, it is necessary to prepare the structure of the protein. Four different human structures of CDC7 kinase were available at the time of the study in the Protein Data Bank (PDB) (see Materials and Methods)^22^. Thus, all of them were selected to this purpose. Once the different structures of the protein were prepared, fpocket analysis was performed. As result, the software detected 22 different cavities in all the structures excepting 4F9B with 21 identified pockets.

**Figure 2.**
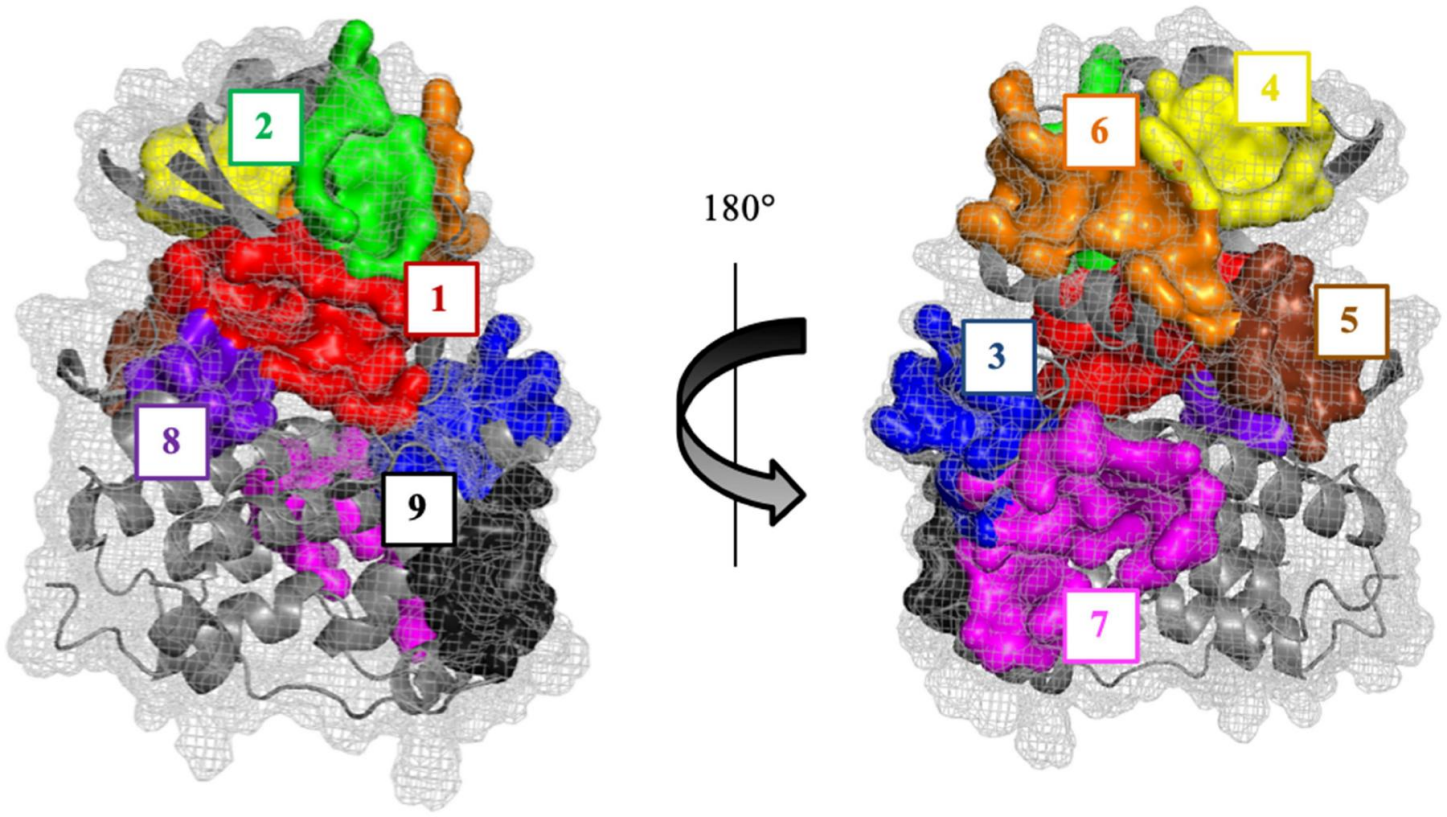
Representative CDC7 structure with the most conserved and druggable cavities found by Fpocket.

Then, all the cavities detected were analysed by visual inspection considering the frequency as they appear and the pocket score (druggability prediction score) given by the program. Once all the cavities were analysed, we observed that only some of these sites were presented in all the CDC7 structures and others were not conserved among them. Finally, nine pockets were found to appear in all the different structures of CDC7 that can be considered as druggable binding sites (Figure 2), although the size and position of each site changes among the different structures and, in some cases, they were detected as two or three different cavities by the program (Table 1).

**Table 1.** Druggable binding sites. Numbering refers to the priority given by the fpocket. Two or more numbers indicate that the overall pocket is detected as different two or more cavities.

Druggable binding sites	Structures (PDB codes)			
	4F99	4F9A	4F9B	4F9C
1	1	1	1	1, 4
2	2	3	2	2
3	3	2	5	6, 21
4	4	6	3	5
5	6	5	7	7
6	7	11, 13	15	13, 15
7	8, 10	7, 9, 12	6, 9	6, 10
8	11	8	8	8
9	18	10	18	18

**Figure 3.**
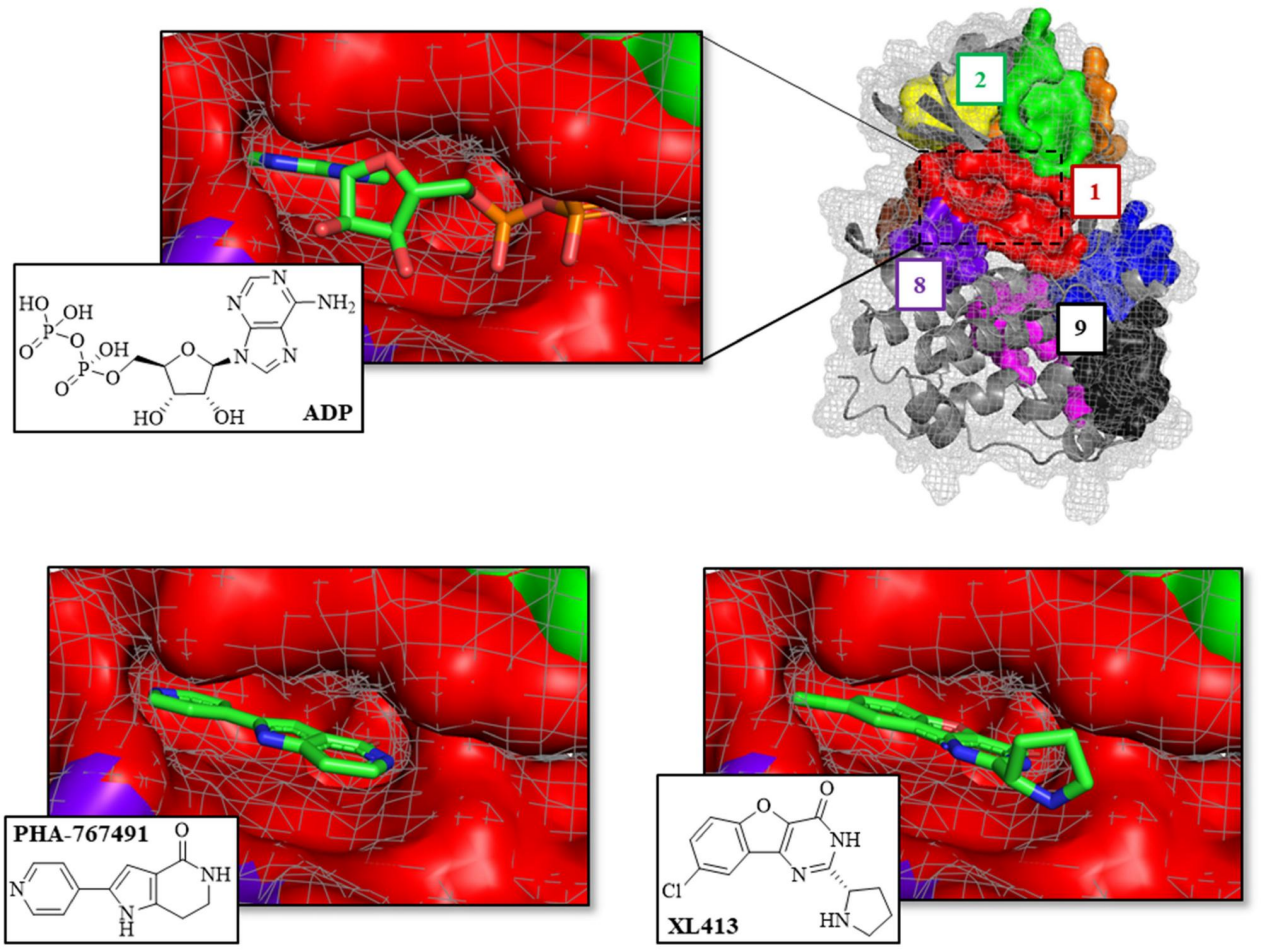
Superposition of the fpocket results with the human CDC7 crystal structure in complex with ADP (PDB code: 4F9A), PHA-767491 (PDB code: 4F9B), and XL413 (PDB code: 4F9C) inhibitors.

As shown in Table 2, the best ranked pocket for all the structures is the cavity 1, corresponding to the catalytic site. Key aminoacidic residues conforming this pocket are collected in Table S1. In the case of 4F9C structure, this pocket was detected as two independent cavities (numbered as 1 and 4). To confirm that pocket 1 corresponds to the catalytic site, the fpocket results and the crystallographic structures of CDC7 with ADP and ATP-competitive inhibitors PHA-767491 and XL413 were superposed. As result, we can confirm that cavity 1 corresponds to the CDC7 catalytic site (Figure 3).

**Table 2.** Docking score and MMGSBA energy values of CDC7 control inhibitors in different surface pockets.

Compound	Catalytic site		Allosteric site 2		Allosteric site 6	
	Docking Score	MMGSBA (Kcal × mol^−1^)	Docking Score	MMGSBA (Kcal × mol^−1^)	Docking Score	MMGSBA (Kcal × mol^−1^)
XL-413	−3.969	−46,93	−2.269	−39,19	−1.721	−39,07
PHA767491	−7.903	−40,19	−3.668	−31,20	−2.271	−32,32
Clofoctol	−3.088	−30,53	−1.515	−42,40	−5.153	−52,20

**Figure 4.**
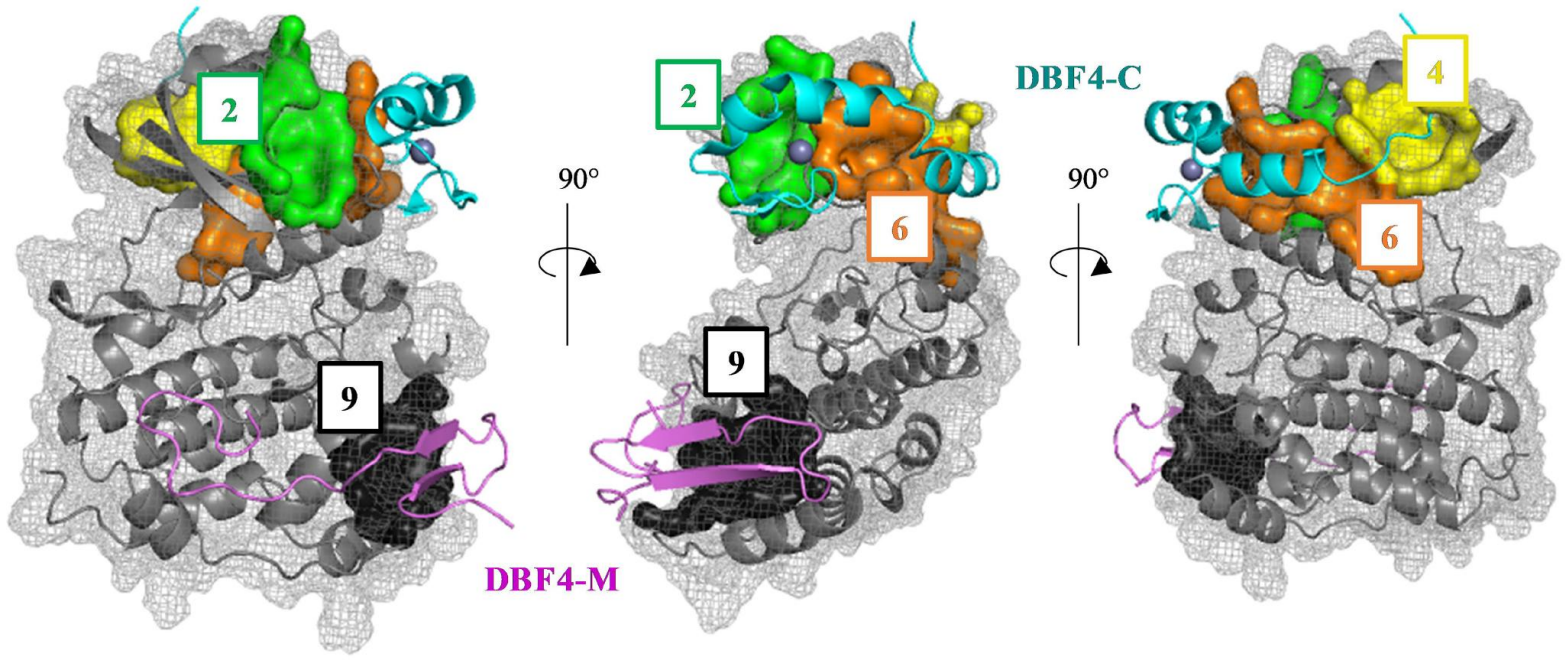
Superposition of the pockets 2, 4, 6, and 9 with the human CDC7-DBF4 crystal structure (PDB code 4F9A). The regulatory subunit (DBF4) is represented in pink (motif M) and cyan (motif C).

This superposition also showed that pockets 2, 4, 6 and 9 correspond to the interaction sites between the protein CDC7 and its activator (DBF4), which is necessary for its kinase activity (Figure 4)^32^.

Pocket 2 is scored as the second-best cavity in three out of the four structures studied (Table 2). It locates in the interaction path of the Nα1 helix, and the β4-β5 and β3-αC loops of CDC7 N lobe with the Zn^2+^ binding domain of DBF4 motif C, which is essential for mitotic functions of the protein^33^. Pocket 4 is also located in the protein-protein interaction surface between β1, β2, β3, and β4 strands, as well as the Nα2 helix of CDC7 N-terminal lobe with the hydrophobic tail of DBF4 motif C, being ranked in the top six positions in all the structures. Pocket 6, detected as two different cavities in 4F9A and 4F9C structures (Table 2), comprises the interaction between α3 helix of DBF4 motif C and the αC helix of CDC7 N lobe. The last pocket locates in the interaction surface between CDC7 and DBF4 motif M is pocket 9, despite is not one of the best ranked sites. In this pocket, the β1 strand of DBF4 motif M pairs with the β1 sheet of CDC7 kinase insert 3 (KI-3) and the αEF and αG helices of the CDC7 C-terminal lobe.

The other four conserved sites (pockets 3, 5, 7, and 8) found in all the crystal structures of CDC7 are located in different sites of the protein but they do not participate in the direct interaction with the regulatory subunit DBF4. Main virtually identified residues involved in all the previous described pockets are reported in the supplementary material (Table S1).

Altogether, and using fpocket program, we have identified nine conserved sites in the surface of CDC7 that can be considered as druggable binding sites. One of these cavities, named pocket 1, corresponds to the catalytic binding site (Figure 3), while the others may be considered as allosteric potential binding sites.

### Pocket selection and virtual screening

Once we have identified the conserved sites on the surface of CDC7 that can be considered as druggable binding sites, the next step was to select the most interesting pockets to search for allosteric modulators.

Although DBF4 motifs M and C are sufficient and necessary to interact with CDC7 kinase and stimulate it^34^, motif C is essential for the activity of the protein by binding to and stabilising its canonical αC helix through its interaction with the DBF4 α3 helix and DBF4 Zn^2+^ binding domain^22^. Meanwhile, the role of motif M may be stabilising linkage between CDC7 and DBF4, allowing the kinase to be switched on and off by disengagement of motif C without full dissociation of the complex.

**Figure 5.**
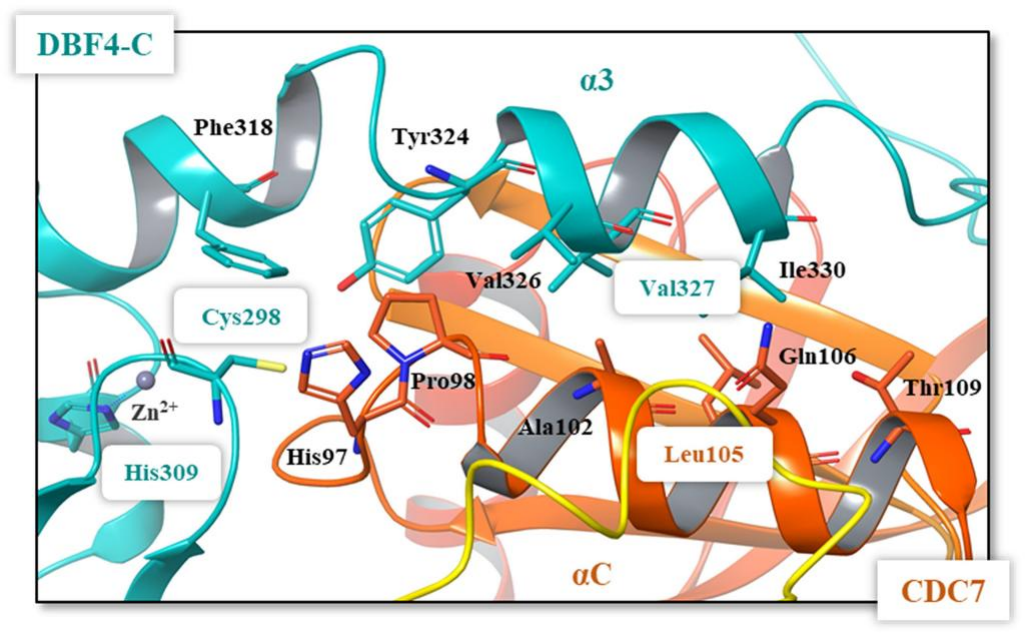
Details of the interface between the DBF4 motif C (green) and the αC helix of CDC7 N lobe (orange). Most important residues are represented in colours.

Among the potential residues involving the interface between CDC7 αC helix and DBF4 motif C, the most important are Cys298 y Val327 as site directed mutations within these residues grossly impaired the kinase activity. The side chain of DBF4 Cys298 is part of the Zn^2+^ binding domain and inserts into a small hydrophobic cavity located at the beginning of the CDC7 αC helix, while DBF4 Val327 is involved in the packing of DBF4 motif C α3 with CDC7 αC helix (Figure 5)^22^. In addition, mutations in DBF4 His309 leads to the disruption of the Zn^2+^ binding domain and reduces drastically the kinase activity. Similarly, site directed mutations within Val327 of DBF4 and Leu105 of CDC7 reduce bioluminiscence signals in the Rluc-PCA assay^19^. Pockets 2 and 6 comprise the interaction site between the canonical αC helix of CDC7 and the DBF4 Zn^2+^ binding domain and DBF4 α3 helix (Figure 5, TableS1), and thus, these two pockets were selected to look for new molecules able to interrupt the interaction between both proteins, modulating the activity of CDC7.

**Figure 6.**
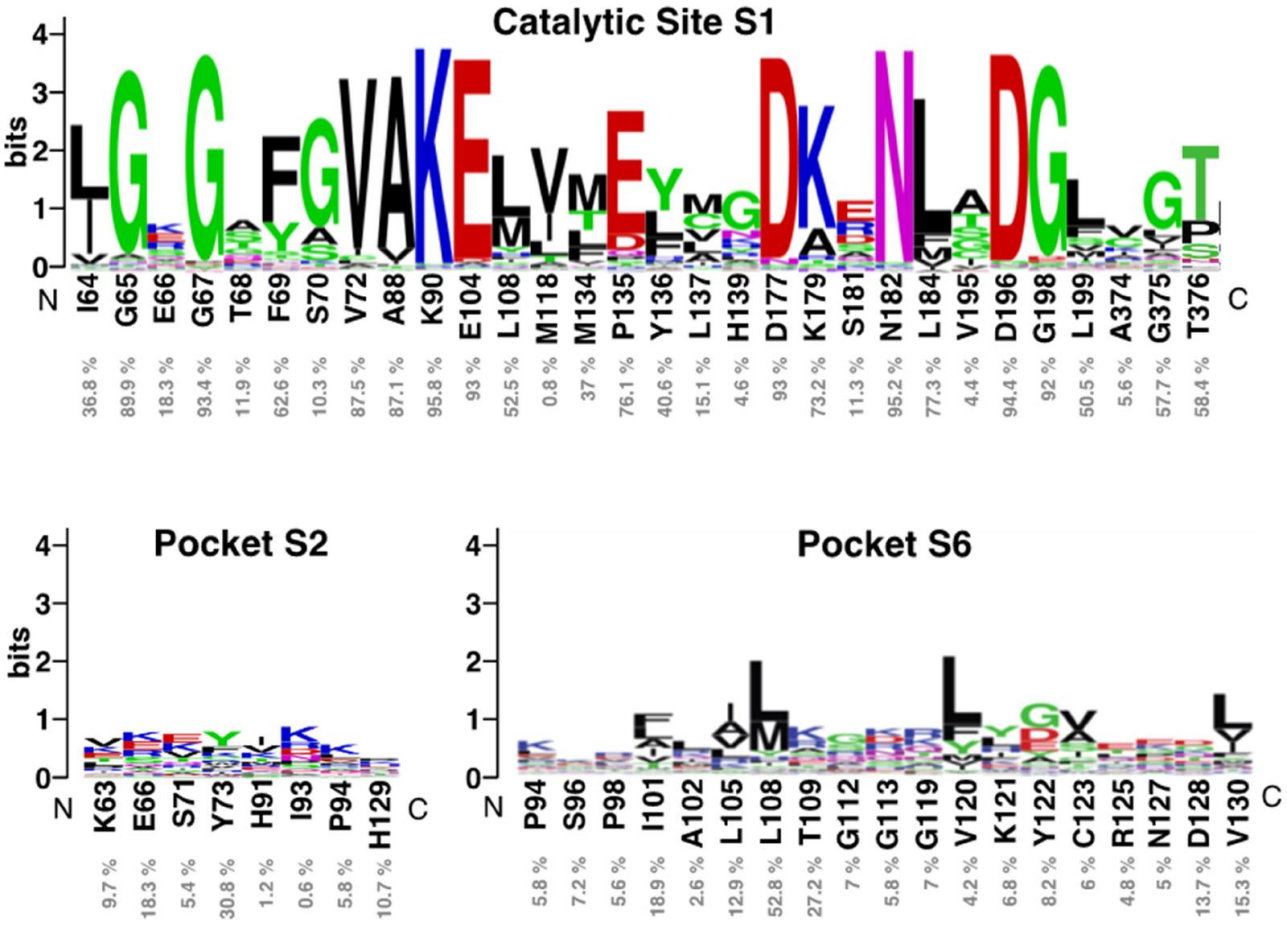
Sequence logo analysis of pockets 1 (catalytic site), 2 and 6 using the multiple sequence alignment (MSA) of 497 human protein kinome reported in 2019^35^. Logos were obtained with the WebLogo server^40^. Each logo consists of stacks of symbols, one stack for each position in the MSA. The overall height of the stack indicates the sequence conservation at that position, while the height of symbols within the stack indicates the relative frequency of each amino at that position, indicated as % below CDC7 residues.

To study how conserved are pocket 1 (catalytic site), as well as pockets 2 and 6, we use the structurally-validated multiple sequence alignment of 497 human kinome reported in 2019^35^. In the Figure 6 the consensus logo is shown, and it can be observed that the pocket 1 is highly conserved because is the catalytic site. However, the pockets 2 and 6 are not well conserved among the 497 human kinase proteins, only residues Tyr73 (pocket 2) and Leu108 (pocket 6) present a percentage of sequence identity greater than 30% among all the kinases. Thus, we can conclude that pockets 2 and 6 are ideal for the design and/or identification of new allosteric inhibitors selective for CDC7.

As source of chemical compounds, our in-house MBC chemical library^20^ was selected it contains 1096 compounds with diverse heterocyclic scaffolds and drug-like properties. All the containing compounds have been synthesised in our laboratories as potential drugs candidates for different medicinal chemistry projects on neurological and infectious diseases while the purity of all of them is >95% by HPLC. MBC library has provided excellent results in hit identification using both phenotypic and virtual screening procedures^36^^,^^37^. It is in continuous growing, now with 2577 compounds, being a very useful tool to find new drug candidate molecules^38^.

Virtual screening of our MBC chemical library in pockets 2 and 6 were performed by docking simulations using the Glide software through Maestro interface (see Material and Methods)^26^ on human CDC7 crystal structure 4F9A. This 3D-structure was selected because pocket 6 was better identified by fpocket. For docking and virtual screening validation, the known ATP-competitive CDC7 inhibitors (PHA-767491 and XL413)^22^ and the allosteric modulator clofoctol^19^ were used. Then, molecular mechanics energies combined with the generalised Born and surface area (MM-GBSA) calculations were performed to re-score the docking results. These methods have been widely used to post-processes docking poses and predict binding affinities of small ligands to biological macromolecules^29^^,^^39^.

Following this procedure, we observed that the control inhibitors PHA-767491 and XL413 showed better binding free energies in pocket 1 than in the two allosteric ones, which is in line with experimental results. In contrast, clofoctol had better binding energies in the allosteric pockets 2 and 6 than in the catalytic one (Table 2). These results validated our docking protocol, which has been applied to the virtual screening step.

**Figure 7.**
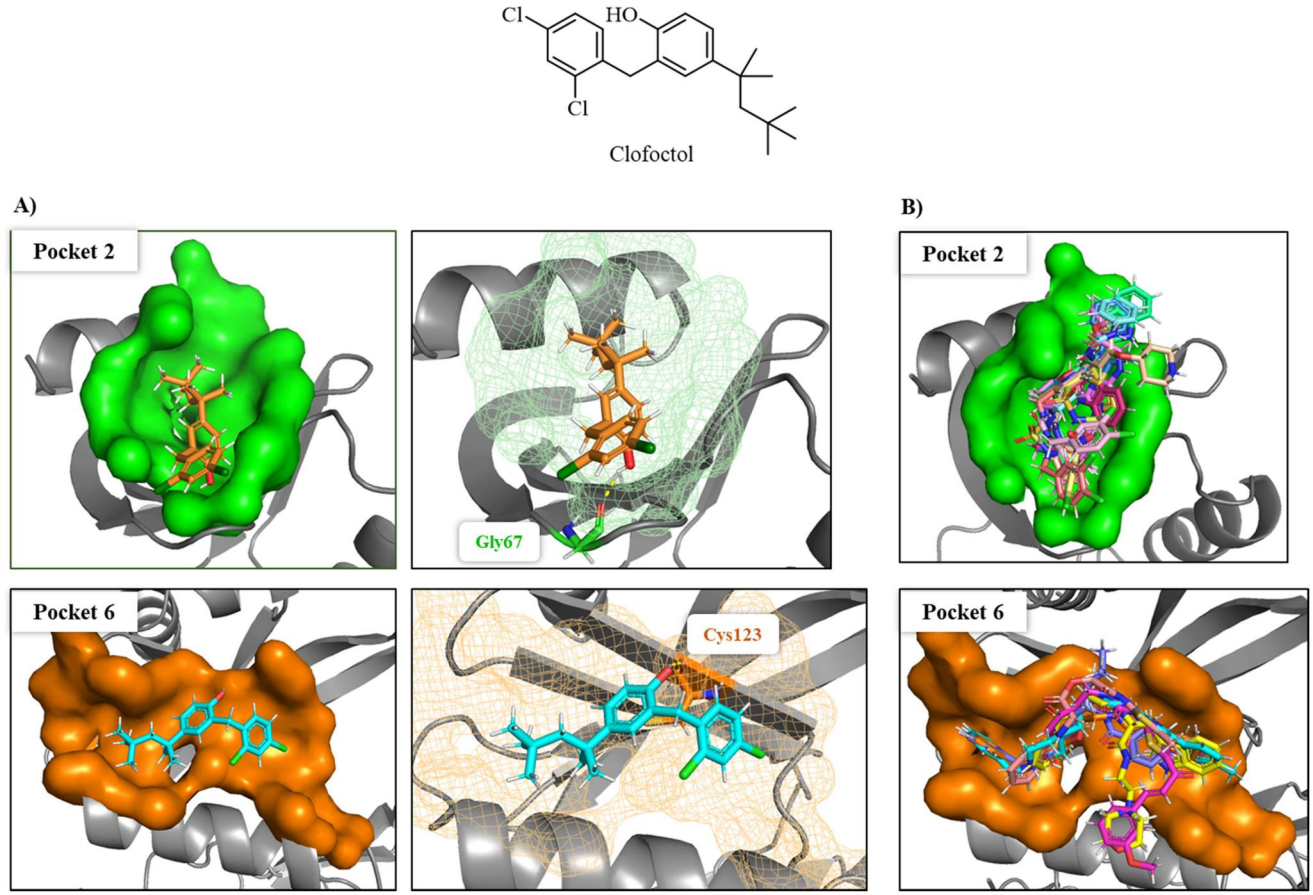
A) Superposition of clofoctol in pockets 6 and 2; B) virtual screening results of MBC library in these two pockets.

Visual inspection of clofoctol poses in pockets 2 and 6, showed better fitting in the allosteric site 6 as corresponds with its MMGSBA energy value. Furthermore, clofoctol in the allosteric pocket 6 occupied the site of the α3 helix of DBF4 motif C binding to the protein through a hydrogen bond with backbone of Cys123 (Figure 7A). This hydrogen bond restriction was also included in the selection of final compounds coming from the virtual screening (Figure 7B).

Finally, the selected molecules in pockets 2 and 6, those with better docking scores than clofoctol in sites 2 and 6 (Figure 7B) were also docked in CDC7 catalytic site (pocket 1) to discard those molecules with a better binding free energy (ΔG_bind_) in this cavity against pockets 2 and 6 trying to avoid catalytic inhibitors. Chemical structures and binding energy values of potential allosteric modulators finally identified by virtual screening are depicted in Table 3, finding a great structural diversity among them.

**Table 3.** Selected molecules from virtual screening as allosteric modulators of CDC7.

Compound	Chemical structure	Allosteric site 2		Allosteric site 6	
		Docking Score	MMGSBA (Kcal × mol^−1^)	Docking Score	MMGSBA (Kcal × mol^−1^)
SC652	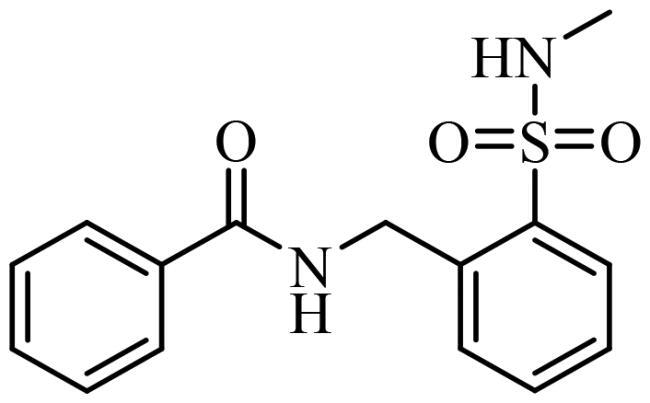	−5.354	−54,80	*	*
JAR3.29	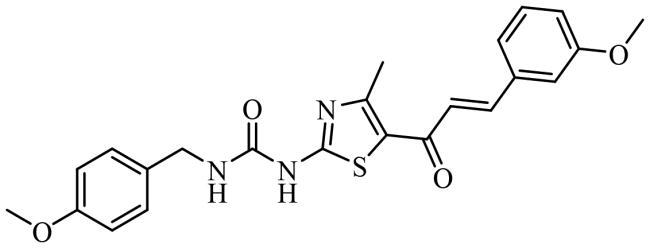	−4.430	−53,18	−4.275	−60,65
MR4.39	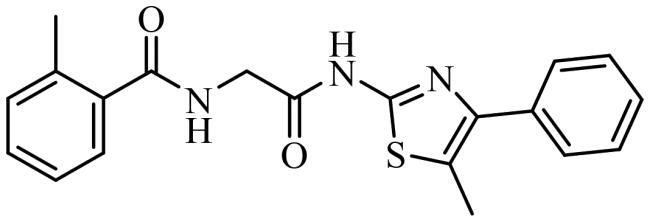	−5.328	−52,59	*	*
VP1.62	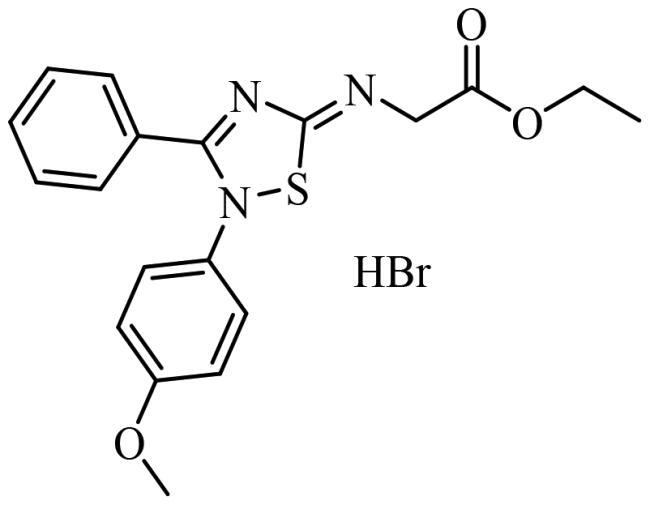	−4.638	−52,55	−3.768	−48,18
SMP2.13	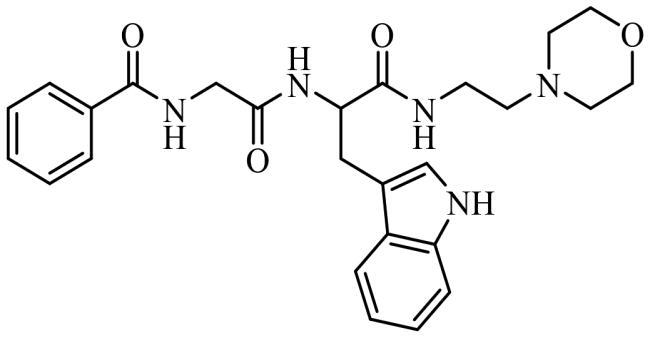	−5.490	−52,37	−5.342	−55,52
MR4.40	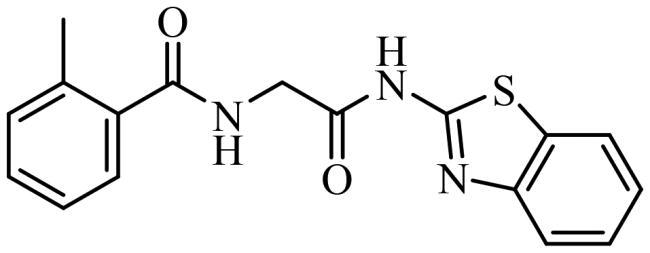	−5.466	−52,16	*	*
AGR1.230	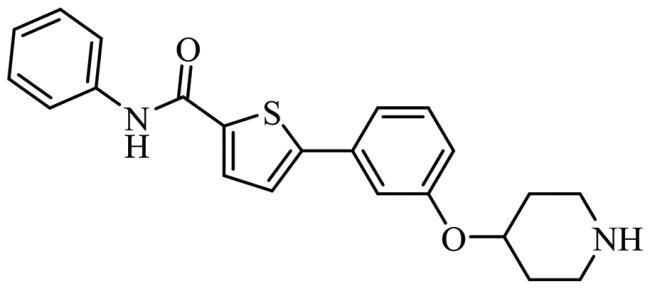	−4.592	−51,54	*	*
AGR1.121	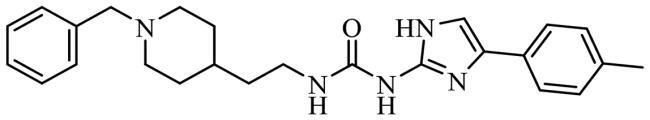	*	*	−4.373	−61,39
Clofoctol	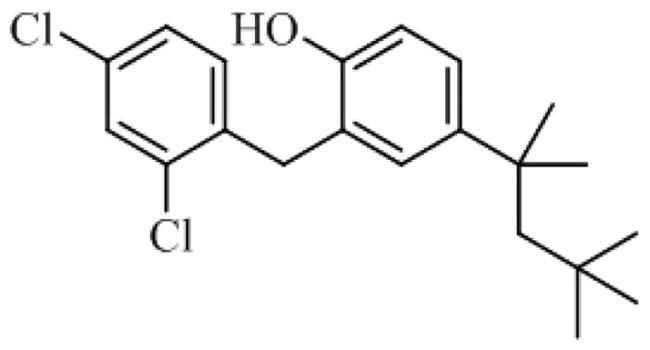	−1.515	−42,40	−5.153	−52,20

* Values not calculated, as the compound did not pass the virtual screening filters in the present pocket.

### 3.3. Experimental Biological evaluation

**Figure 8.**
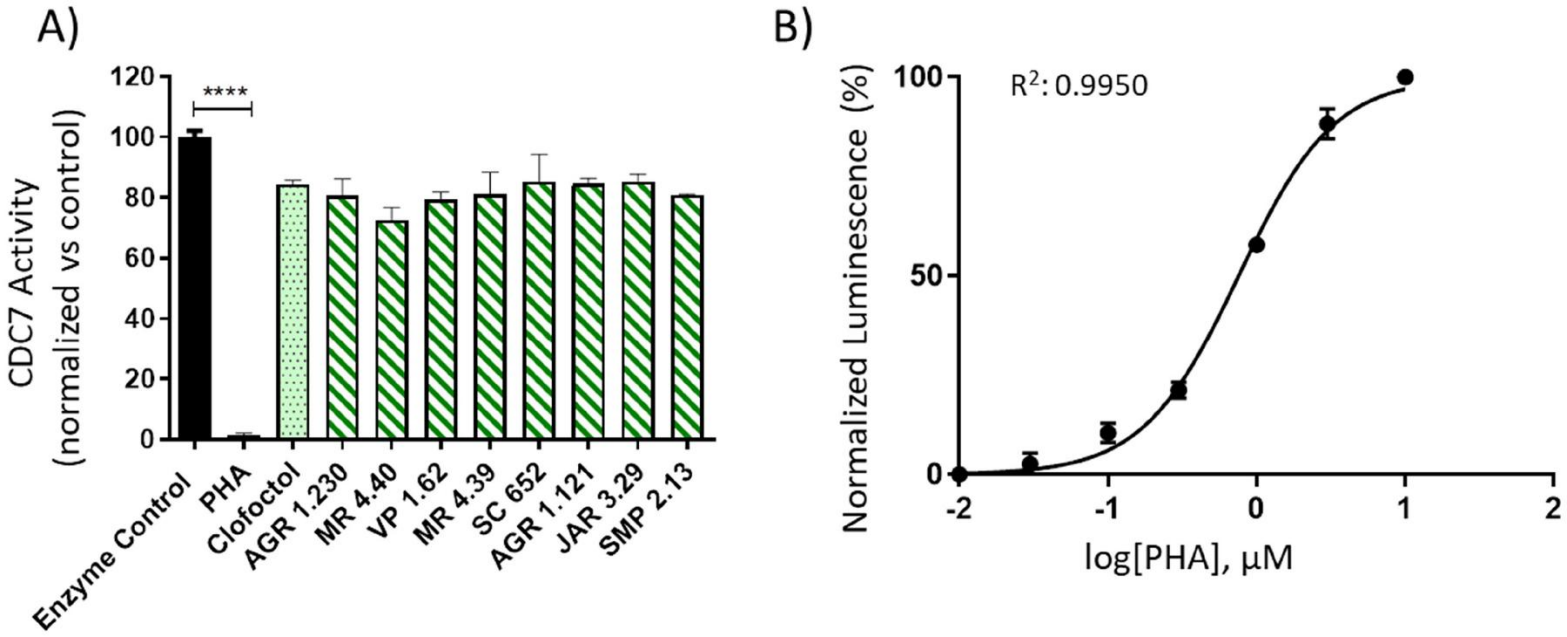
Kinase-Glo assay was used to screen compounds for activity against CDC7-DBF4 kinase. A) CDC7 activity in the presence of compounds at 10 uM. Clofoctol (allosteric inhibitor, 20 uM) and PHA (ATP-competitive inhibitor, 10 uM) were used as controls. B) Graph represents the IC_50_ of PHA. The activity is proportional to the difference of the total and consumed ATP. The inhibitory activities were calculated based on maximal activities measured in the absence of inhibitor. Bars are the mean ± SD of three independent experiments. Statistical analysis was performed using One-way ANOVA followed by Bonferroni’s post-test (****p < 0.0001).

With the aim to confirm experimentally the allosteric modulators identified, we performed several different approaches. In the first one, and to discard compounds able to bind to the ATP cavity, a kinase inhibition using human recombinant CDC7-DBF4 enzyme and Kinase-Glo methodology was used (Figure 8). In that case, PHA-767491 (ATP-competitive inhibitor) and clofoctol (allosteric inhibitor) were used as control references. An IC_50_ value of 0.73 µM was calculated for PHA-767491 (PHA) (Figure 8B). However, none of the 8 compounds here tested at the concentration of 10, 25 and 50 µM nor clofoctol at the effective dose described (20 µM)^19^ showed any activity (Figure 8A, Figure S1). These results may be explained as the *in vitro* interaction between CDC7 and its activator DBF4 is difficult to be broken once it is formed and all these compounds have low binding affinity to the catalytic site.

We next evaluated the potential activity of these new drugs in a cell-based model, specifically in the human neuroblastoma SH-SY5Y cell line, were DBF4 subunit binds to CDC7 when DNA replication is going to take place. CDC7 plays an important role in the initiation of DNA replication during the G1/S transition of the cell cycle, as it phosphorylates the helicase complex known as minichromosome maintenance complex (MCM), essential for DNA unwinding and therefore DNA replication^9^.

In order to evaluate the ability of the studied compounds in delay cell cycle progression, a bromodeoxyuridine (BrdU) incorporation assay was performed. Cells were treated with PHA, clofoctol or the studied compounds for 24 h. After a BrdU pulse, the treated cells were fixed and dual-stained with anti-BrdU and 7-AAD, prior to flow cytometry analysis.

**Figure 9.**
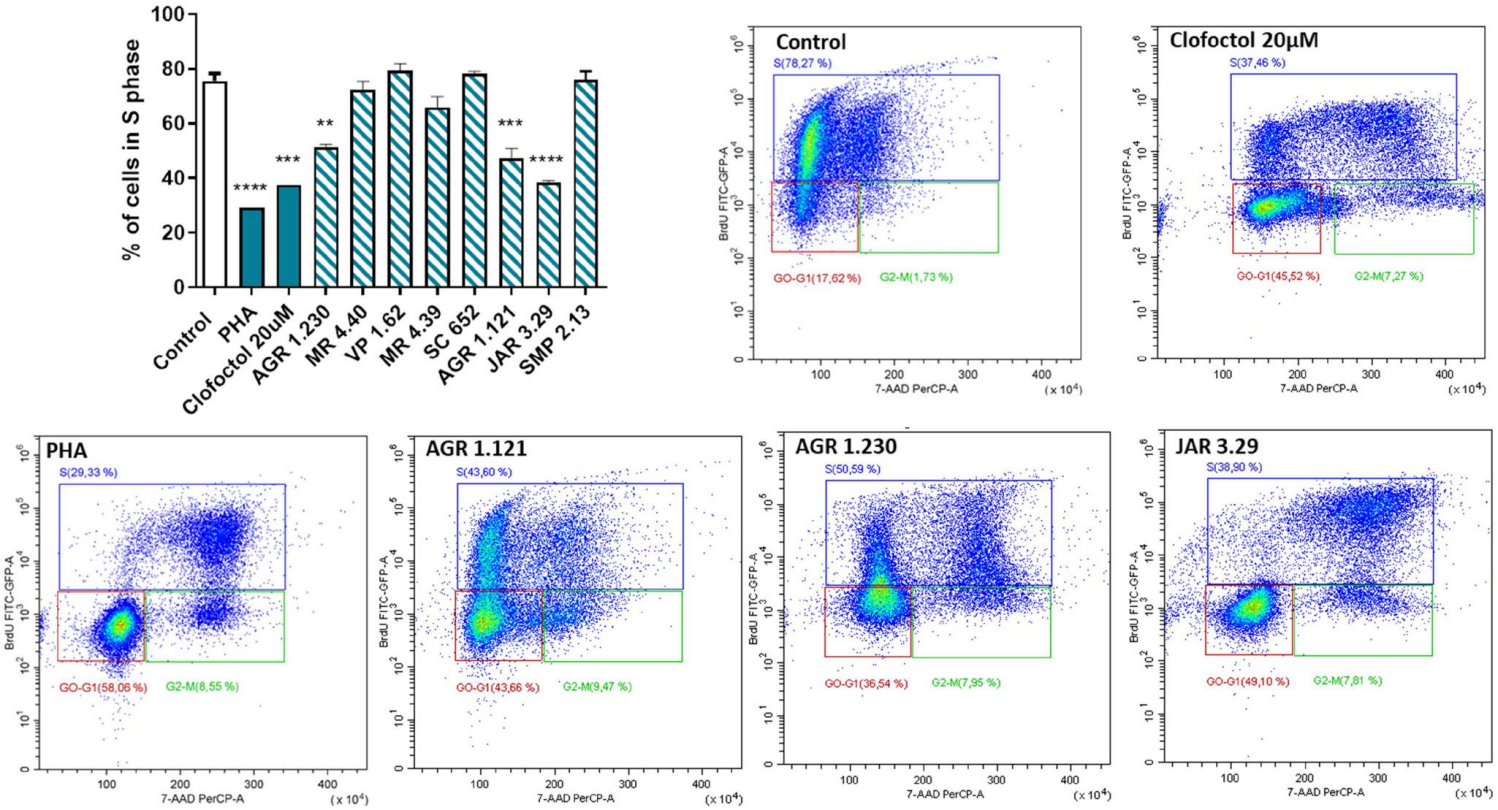
Compounds effect on cell cycle distribution shown by BrdU incorporation assay in SH-SY5Y cell line. Cells were treated with Clofoctol, PHA and studied compounds (10 µM) for 24h. The treated cells were pulsed with 20 μM BrdU in culture medium 4h after compounds treatment and conducted by FITC-BrdU Flow Kit. Graph represents the percentages of cells in S phase. FACS analysis was performed to demonstrate cell cycle distribution: G0/G1 phase (red region) S phase (blue region) and G2/M phase (green region). Plots of positive compounds are shown (BrdU: Y-axis and 7-AAD: X-axis). Bars are the mean ± SD of three independent experiments. Statistical analysis was performed using One-way ANOVA followed by Bonferroni’s post-test (**p < 0.01; ***p < 0.001; ****p < 0.0001).

Results showed that clofoctol, PHA and only compounds AGR 1.230, AGR 1.121 and mainly JAR 3.29 significantly block DNA replication (S phase) and delay cell cycle progression leading to the accumulation of cells in the G0/G1 phase (Figure 9, Figure S2). Therefore, the significant decreased in the percentage of cells in the S phase of the cell cycle shown after the treatment with AGR 1.230, AGR 1.121 and JAR 3.29 may be due to the inhibition of CDC7-DBF4 interaction, which avoids the activation of CDC7 after the BrdU pulse (Figure 9). The higher activity found in JAR3.29 may be also explained considering that this heterocyclic drug candidate is able to effectively bind to allosteric pockets 2 and 6 and probably interrupting better the protein-protein interaction between CDC7 and its activator DBF4.

## Conclusions

Altogether, eight new druggable cavities on CDC7 surface for potential allosteric modulation have been identified using the free open source program fpocket. Among these pockets, four of them are located in different interaction sites between the kinase and its activator (DBF4) defining the interacting key residues. Moreover, we carried out virtual screening of our in-house MBC chemical library to identify novel allosteric inhibitors of pocket 2 and 6 with the goal to interrupt the CDC7-DBF4 motif C protein-protein interaction essential for the kinase activity. Several compounds with great structural diversity have been selected for further biological studies. Compounds AGR 1.230, AGR 1.121 and JAR 3.29 have shown to be able to inhibit CDC7 in a cellular model in the same way as clofoctol does, corroborating the possibility to modulate allosterically CDC7 by interrupting the protein-protein interaction with DBF4 motif C. These compounds may represent new potent pharmacological tools for different therapeutic studies where the activity of CDC7 is involved, mainly in cancer and TDP-43-proteinopathies such as ALS.

## Supplementary Material

Supplemental Material
