# Supplementary material for: Druggable cavities and allosteric modulators of the cell division cycle 7 (CDC7) kinase
Source: J Enzyme Inhib Med Chem. 2024 Jan 11;39(1):2301767. doi: 10.1080/14756366.2024.2301767 (PMC10786434; doi:10.1080/14756366.2024.2301767)

REVISED 26.11.2023

Supporting information

Druggable cavities and allosteric modulators of the cell division cycle 7 (CDC7) kinase

Table S1. Main aminoacids involved in the druggable pockets found on CDC7 surface[a]

Pocket	description	Key aminoacids
1	Catalytic site	Ile64, Gly65, Glu66, Gly67, Thr68, Phe69, Ser70, Val72, Ala88, Lys90, Glu104, Leu108, Met118, Met134, Pro135, Tyr136, Leu137, His139, Asp177, Lys179, Ser181, Asn182, Leu184, Val195, Asp196, Gly198, Leu199, Ala374, Gly375, and Thr376
2	Zn^{2+} binding domain of DBF4 motif C	Asp43, Lys46, Leu47, Ala50, Lys63, Glu66, Ser71, Tyr73, His91, Ile93, Pro94, and His129
4	the hydrophobic tail of DBF4 motif C	Val57, Phe58, Ala77, Gln78, Leu79, Gln80, Ile87, Lys121, Tyr122, Phe124 and, Ala133
6	α3 helix of DBF4 motif C	Pro94, Ser96, Pro98, Ile101, Ala102, Leu105, Leu108, Thr109, Gly112, Gly113, Gly119, Val120, Lys121, Tyr122, Cys123, Arg125, Asn127, Asp128, and Val130
9	β1 strand of DBF4 motif M	Thr386, Leu421, Leu424, Met428, Lys445, Ser446, Ile447, and Leu448
3		Arg176, Gln201, Gly202, Thr203, His204, Pro389, Asn390 and Gln391
5	behind the catalytic loop	Gly113, Gln114, Asp115, Asn116, Val117, Met118, Gly119, Pro135, Tyr136, Leu137, Lys190, Lys191, Tyr192, and Ala193.
7		His169, Gln170, Gly172, Thr203, His204, Asp205, Lys207, Glu383, Cys388, Asn390, Gln391, Thr392, Thr393, Ala394, Met397, Thr442, Phe443, Asn553, Pro554, Ala555, Arg557, Ile558, Thr559, and Ala560
8	Located at the entrance of the catalytic site	Ile64, His139, Glu140, Ser141, Phe142, Leu143, Ile145, Tyr157, Pro180, Ser181, Phe183, Leu184, Tyr185, and Tyr412

[a] These predicted aminoacids should be only experimentally confirmed by site-directed mutagenesis

Figure S1. CDC7 activity in the presence of compounds at 25 and 50 uM. Clofoctol (allosteric inhibitor, 20 uM) and PHA (ATP-competitive inhibitor, 10 uM) were used as controls. Kinase-Glo assay was used to screen compounds for activity against CDC7-DBF4 kinase. The activity is proportional to the difference of the total and consumed ATP. Bars are the mean ± SD of three independent experiments. Statistical analysis was performed using One-way ANOVA followed by Bonferroni's post-test (****$p < 0.0001$).

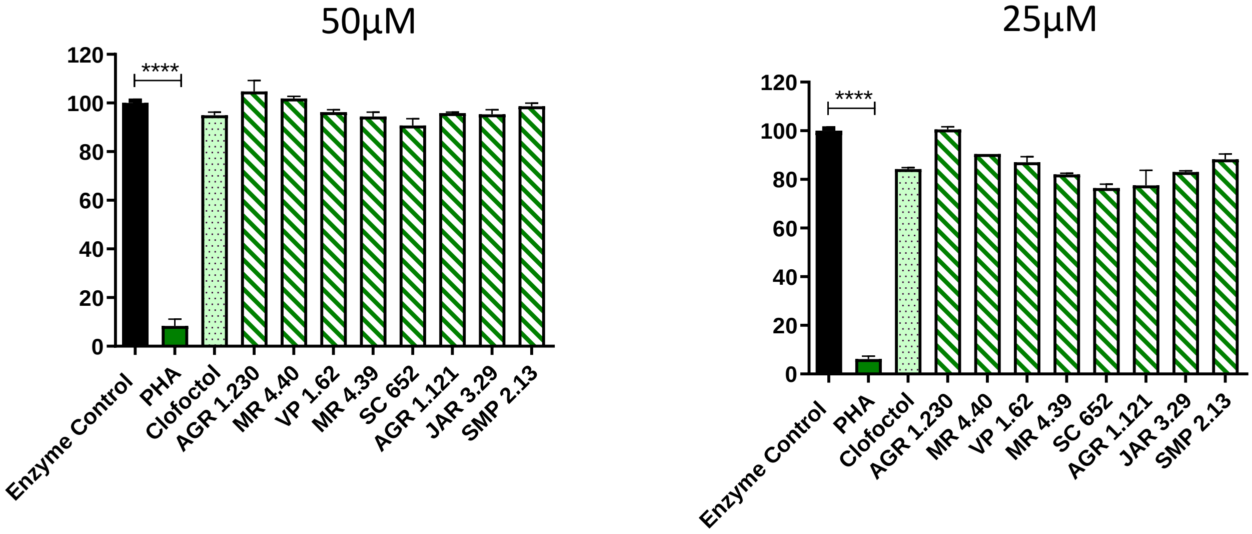

Figure S2. Compounds effect on cell cycle distribution shown by BrdU incorporation assay in SH-SY5Y cell line. FACS analysis was performed to demonstrate cell cycle distribution: G0/G1 phase (red region) S phase (blue region) and G2/M phase (green region). Plots shown BrdU in Y-axis and 7-AAD in X-axis.

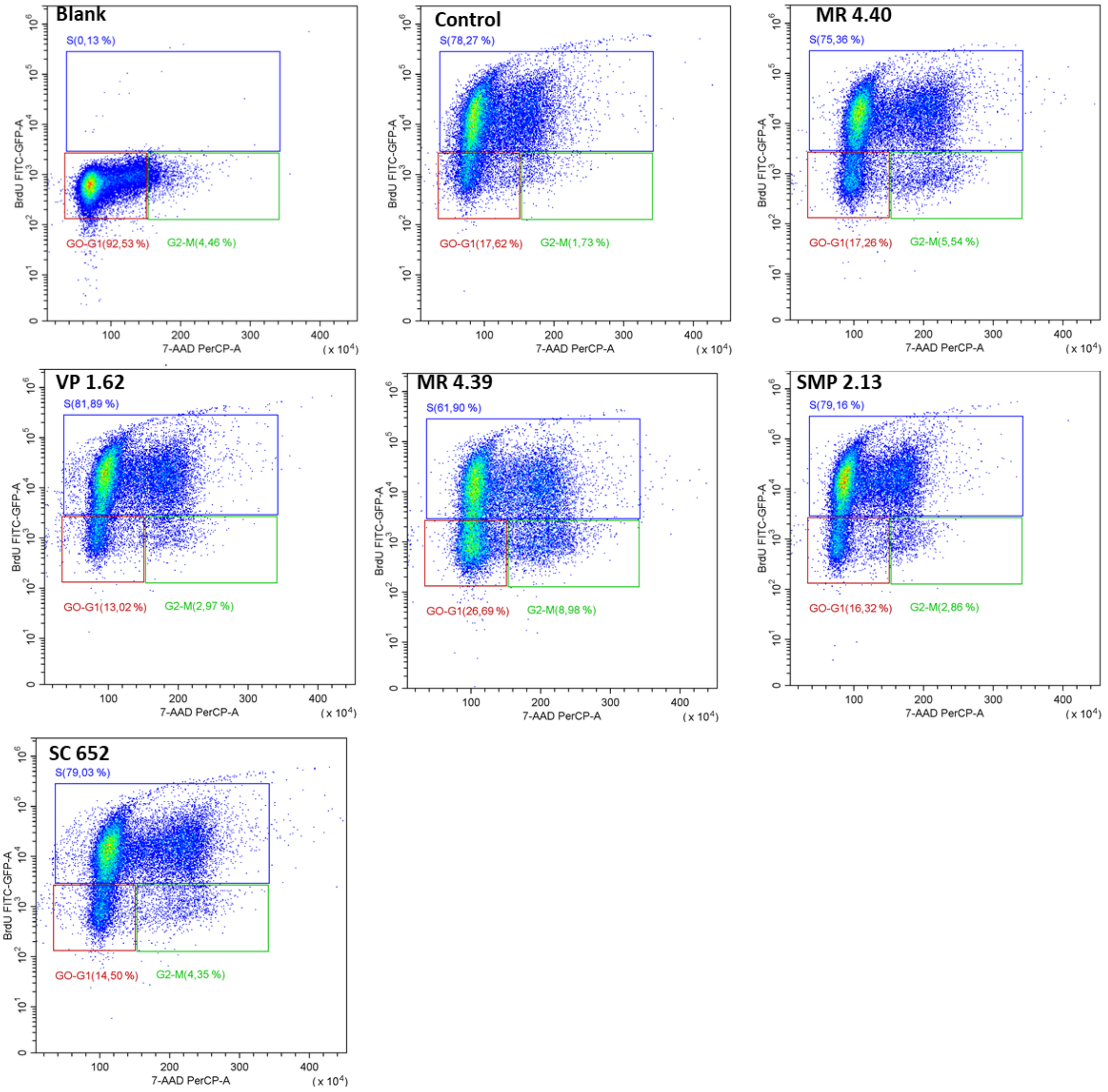

Supplement: Supplemental Material [file IENZ_A_2301767_SM1146.pdf]
